# How to Avoid Colds

**Published:** 1896-02

**Authors:** 


### How to Avoid Colds.

There is one simple way of avoiding colds—keep your mouth shut while out of doors. The man or woman who comes out of an overheated room, especially late at night, and breathes through the mouth, will either catch a bad cold or irritate the lungs sufficiently to cause annoyance and unpleasantness. If people would just keep their mouths shut and breathe through their noses, this difficulty and danger would be avoided. Chills are often the result of people talking freely while out of doors just after leaving a room full of hot air, and theater-goers who discuss and laugh over the play on their way home are inviting illness. It is, in fact, during youth that the greater number of mankind contract habits or inflammation which make their whole life a tissue of disorders.—*Family Doctor.*

MINISTER—And so you think you are to be a minister when you grow up, my little man?

LITTLE MAN—Yessir. Mother says I'm just cut out for a minister.

MINISTER—Because you do so love to be good?

LITTLE MAN—No; because I'm always gettin' sore throat, and bein' ordered away for my health.—*Ex.*

